# Evidence Based Management of Acute Heart Failure in the Era of COVID-19 Pandemic

**DOI:** 10.1007/s44231-022-00003-6

**Published:** 2022-05-23

**Authors:** Lexin Wang

**Affiliations:** Wagga Medical Centre, 4 Baylis Street, Wagga, NSW 2650 Australia

**Keywords:** Acute heart failure, COVID-19, Coronary artery disease, Angiotensin converting enzyme inhibitors, Telehealth, cardiovascular health

## Abstract

This editorial is to highlight current issues of heart failure management during COVID-19 pandemic.

## Introduction

Heart failure is a significant health burden that affects all societies. More than one in ten people over the age of 75 will develop heart failure, and the overall incidence of heart failure in women and men aged 55 years and above is 29% and 33%, respectively, in their lifetime [1]. Acute heart failure (AHF) refers to a complex syndrome where there is an acute onset of symptoms of heart failure that requires immediate intervention, whereas chronic heart failure often describes those who have had symptoms of heart failure for a period of at least several months. There has been a variety of reported causes for heart failure and the most common ones being coronary artery disease, cardiomyopathy, and hypertension. AHF is associated with a high rate of morbidity and mortality, and significant health care costs, as patients with AHF often require several stages of treatment, which involves emergency department visits, hospitalisation, discharge planning and continuing treatment at home or in community settings.

The pandemic of coronavirus disease 2019 (COVID‐19) has become one of the greatest challenges to the public health around the globe. At the time of the writing, there were 281 million confirmed cases of COVID-19 globally, with 5.4 million COVID-19 related deaths. COVID-19 mainly affects the respiratory system, but it also has severe impact on cardiovascular and metabolic systems, resulting in multiple organ failure [2, 3]. In patients who were admitted to hospitals for COVID-19 related conditions, almost one third showed signs of chronic and acute myocardial injury [4]. A recent systemic review has found that, following COVID-19 infection, the incidence of AHF was 2%, myocardial infarction was 4%, angina was 10%, and arrhythmia was 18% [5]. It also revealed that COVID-19 infection was associated with 25% incidence of venous thromboembolism and 19% of pulmonary embolism [5]. In hospitalized COVID-19 patients who had no history of heart failure, one in four was found to have new onset of heart failure.

## COVID-19 Infection and Heart Failure

The mechanisms by which COVID-19 leads to cardiac injuries are yet to be delineated, and the long-term impact of COVID-19 on cardiac function and survival remains to be seen. It is likely that systemic and myocardial inflammation following COVID-19 infection is the main cause of myocardial injuries, such as elevated plasma troponin levels, angina or ST elevation on ECG. Only a small portion of patients with angina or ST elevation after COVID-19 infection have clinically significant stenosis on coronary angiogram [6]. The increased venous thromboembolic events following COVID-19 exposure, such as pulmonary embolism [5], also impose pressure on the right ventricle, leading to right ventricular dysfunction. Myocardial injuries are more likely to develop in patients who have a history of coronary artery disease, atrial fibrillation, or heart failure [7]. After COVID-19, patients with multiple comorbidities are more susceptible to new onset of heart failure or deterioration of heart failure symptoms [8]. Acute or chronic cardiac injuries from COVID-19 infection were strongly associated with impaired survival at six months of infection [4]. Patients with impaired ventricular function or heart failure are more likely to have a poorer prognosis than those with normal cardiac function after COVID‐19 infection [9, 10].

## Management of Acute Heart Failure

Investigation and treatment of precipitating factors for AHF is essential. Acute coronary syndrome, severe hypertension, arrhythmias, mechanical catastrophes, such as ruptured ventricular septum, acute valvular regurgitation, or pulmonary embolism, must be identified and treated [11]. Cardiogenic shock and acute respiratory failure should be managed as a matter of urgency. Pharmacotherapy remains the mainstay treatment for AHF. Diuretics, vasodilators, and inotropes are proven pharmacological treatments for AHF. Intravenous inotropic agents, such as dobutamine, may be considered in patients with peripheral hypoperfusion and congestion refractory to other therapies to improve symptoms and end-organ function [11].

Hemodynamic and fluid management in patients with AHF and COVID-19 is critically important. Hypovolemia is common in critical COVID-19 patients due to reduced oral intake or increased fluid loss, or both [12]. Patient’s volumetric status as well as response to fluid replacement should be carefully assessed. Prolonged use of intravenous diuretics must be justified to avoid dehydration. In patients with hemodynamic instability, fluid therapy or administration of vasoactive agents may be guided by non-invasive or invasive monitoring, such as bed-side echocardiography or thermodilution methods [12]. In patients with life-threatening heart failure refractory to pharmacotherapy, extracorporeal life support might be used as a last resort to control the symptoms [12].

Devices, such as non-invasive ventilation or temporary mechanical circulatory support devices, can also provide symptomatic relief for AHF. In patients with severe pulmonary congestion, early application of non-invasive ventilation significantly improves oxygenation and haemodynamic instability in comparison with conventional oxygen therapy [12, 13]. This treatment modality should be considered in all patients with AHF when hypoxaemia and tachypnoeic status were not improved by oxygen therapy.

## Telehealth and Heart Failure Management

The management of heart failure in the setting of COVID-19 has additional challenges. First, delayed care or reluctance of patients to seek medical attention due to self-isolation after contracting COVID-19, or fears of COVID-19 contamination in a clinical setting, may result in progression of stable chronic heart failure to advanced or decompensated heart failure, which can be difficult to manage in a community setting. Patient’s fears of contracting COVID-19 in outpatient clinics or in hospitals are understandable. Frontline healthcare workers may contract COVID-19 from community or from patients presented to the clinics or hospitals. Many hospitals admit COVID-19 and non-COVID-19 patients from the same sites and truly isolating COVID-19 from the non-COVID-19 patients is not always possible [14]. In addition, due to the long intubation periods of COVID-19 infection, many infected patients remain asymptomatic [14]. The combination of these factors increases the risk of COVID-19 transmission in the healthcare setting. Patients with AHF due to delayed presentation and management often require hospital admission. A rise in morbidity or mortality is also likely due to delayed diagnosis or management of heart failure.

In response to this dilemma, in many countries, the usual patterns of care for heart failure in the primary care setting have changed, by extensive use of telehealth or video consultations, with reduced in‐person outpatient visits to minimise the risk of contracting COVID-19 for both patients and health care workers [15, 16]. Telehealth or video calls enable physicians to monitor some of the signs or symptoms of heart failure, such as daily weight, pulse rate, blood pressure or signs of volume overload such as peripheral oedema. Compliance of medications can also be checked through this type of consultations. In this author’s experience, telehealth is particularly welcomed by elderly patients, or by those who have difficulties in accessing transport to physically attend clinics. It is also popular among patients who live in rural or remote areas where physical access to medical facilities is not readily available. Although telehealth appears to be feasible in the management of chronic or stable heart failure, evidence is needed to ascertain the effectiveness of this modality on improving the short- or long-term outcomes. In the setting of AHF, as majority of patients require admission to a hospital for assessment and treatment, telehealth may be best used as a screening tool for patients with deteriorating cardiac function needing admission, or for follow-ups after hospital discharge.

**Fig. 1:**
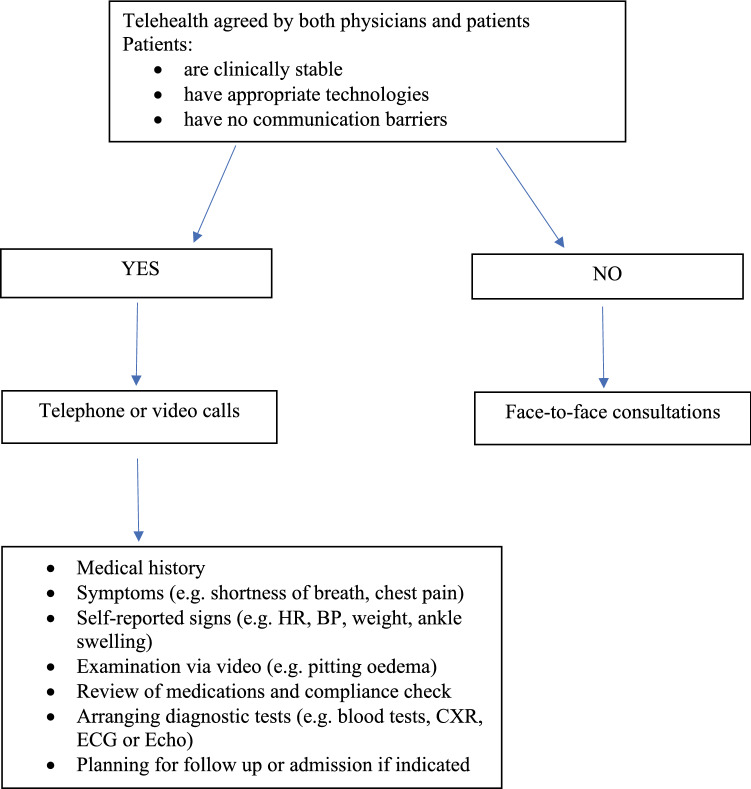
A flow chart of telehealth delivery to heart failure patients in an outpatient setting. *HR* heart rate, *BP* blood pressure, *CXR* chest X-ray, *ECG* electrocardiogram, *Echo* echocardiography

Figure 1 summarises this author’s practice in organising and conducting telehealth in an outpatient setting. This method has been used during COVID-19 pandemic in patients who were self-isolating at home, and in patients who were reluctant to attend the clinics to avoid COVID-19 exposures. The process requires a high level of cooperation among the administrative staff, physicians, patients and their carers. Similar approaches have recently been reported by other investigators [15, 16].

While the COVID-19 pandemic has forced some government organisations and healthcare providers to use more telehealth for the care of heart failure patients, integration of this model of care into day-to-day heart failure management in a post-pandemic era remains a work in progress. Several studies that conducted in a non-pandemic environment have shown that telemonitoring of heart failure patients was feasible, and telemonitoring and telephone support can reduce mortality and heart failure‐related hospitalisations [15, 16]. It is likely that telehealth may be used as an additional tool in a selected group of heart failure patients (such as those who do not need a face-to-face encounter) after the COVID-19 pandemic.

## Angiotensin Converting Enzyme Inhibitors in the Presence of COVID-19 Infection

The use of some of the heart failure medications in the setting of COVID-19 infection needs further discussion. There have been some concerns about the use of angiotensin converting enzyme inhibitors (ACEI) or angiotensin-2 receptor blockers (ARBs) in patients contracted with COVID-19. These concerns were largely based on the ability of COVID-19 virus to bind angiotensin-converting enzyme 2 (ACE2) receptors that are present on the surface of myocytes. There was a believe that the use of ACEI and ARBs may upregulate the ACE2 receptors, which in turn may enhance the myocardial uptake of the COVID-19 virus and the detrimental effect on the heart. However, the interplay between the ACE2 receptors and ACEI or ARBs remains poorly understood, with no clear evidence from available animal or human studies that ACEIs or ARBs lead to over expression of ACE2 receptors in the myocytes. Preliminary observations on patients hospitalized with COVID-19 showed that treatment with ACEI/ARB for hypertension during admission did not change the mortality rate [17]. ACEI/ARBs are well established therapies for hypertension, heart failure and other cardiovascular diseases. Therefore, in the absence of evidence against their use, they should be continued in patients with AHF or other cardiovascular diseases after exposure to COVID-19 [18].

## Conclusions

Heart failure represents one of the most common cardiovascular conditions associated with significant morbidity and mortality. COVID-19 pandemic has made the care for patients with heart failure even more complex. Pre-existing cardiac conditions, such as heart failure, arrhythmia or coronary artery disease, elevate the risk of morbidity and mortality of COVID-19, and COVID-19 itself is associated with serious cardiovascular complications in those with or without a history of cardiac disease. While the traditional pharmacological and non-pharmacological therapies to heart failure remain unchanged in the era of COVID-19 pandemic, new models of cardiovascular health services, such as telehealth, need to be continued or refined to minimise the risk of viral transmission in patients and healthcare providers, and to optimize the outcomes of patients with heart failure.
